# Trends in antibiotic prescribing patterns in sixteen in-center dialysis units

**DOI:** 10.1017/ice.2026.10499

**Published:** 2026-07-06

**Authors:** Ankur Shah, Cheston Cunha, Filipe Monteiro, Erika Dagata

**Affiliations:** 1 Brown University Warren Alpert Medical School, USA; 2 Rhode Island Hospital, USA,; 3 Brown University, Medicine, Providence, USA

## Abstract

**Objective:**

Patients receiving maintenance hemodialysis are frequently exposed to antibiotics, a proportion of which may not be necessary thereby increasing the risk of antibiotic-associated adverse events. Antimicrobial stewardship programs remain underdeveloped in outpatient dialysis facilities. This study aimed to quantify antibiotic prescribing trends across in-center dialysis units and identify patient- and facility-level factors associated with decreased antibiotic use to inform the future stewardship strategies.

**Design:**

Retrospective observational cohort study among 16 in-center hemodialysis units

**Methods:**

Antibiotic prescribing patterns and patient- and unit-level characteristics were analyzed among patients receiving maintenance hemodialysis from April 1, 2021, to March 31, 2024. Antibiotic prescribing trends were analyzed using general linear models. Generalized estimating equations were used to identify factors associated with decreased prescribing.

**Results:**

A total of 937 (26.4%) among 3,549 patients received ≥1 antibiotic dose, with an average of 15.36 doses per 100 patient-months, ranging from 4.82 to 30.87 doses/100 patient months. Types and frequency of antibiotics prescribed among units varied considerably. Trends in antibiotic prescribing did not decrease among 10 (62.5%) of the in-center hemodialysis units. Patient-level characteristics in units associated with a decrease in prescribing included Black race, Hispanic ethnicity, age 56–65, and diabetes as the cause of kidney failure.

**Conclusions:**

There is substantial variability in antibiotic prescribing across dialysis units. Decreasing trends in prescribing were observed in a subset of units and were associated with patient-level factors. These findings support the need for developing and implementing antimicrobial stewardship programs targeting the outpatient dialysis setting.

## Introduction

Although the benefits of antibiotics in the treatment of bacterial infections are undisputed, antibiotic exposure can result in numerous adverse events, including the emergence of antimicrobial-resistant pathogens, *Clostridioides difficile* infections, drug-drug interactions, allergic reactions and end-organ toxic effects.^1^ It is estimated that up to 30% of prescribed antibiotics are unnecessary.^2^ In an effort to improve antimicrobial prescribing and limit unnecessary antibiotic exposure and associated adverse events, the Centers for Medicare and Medicaid Services (CMS) and the Joint Commission require the implementation of antimicrobial stewardship programs in hospitals and nursing home facilities.^3^ Comparable efforts, however, are underdeveloped in outpatient dialysis facilities.

Persons on maintenance hemodialysis are at substantial risk of infections due to an impaired immune system, frequent accessing of the bloodstream during hemodialysis, and use of hemodialysis central venous catheters.^4,5^ As a result, they are frequently exposed to antibiotics with at least one-third receiving antibiotics during one year.^6^ A total of 30%–58% of antimicrobial doses are not indicated in this patient population.^7,8^ Rates of antibiotic prescribing in in-center dialysis facilities are also increasing; among 7,429 facilities reporting to NHSN, the proportion of facilities with an unsupported intravenous antibiotic start increased yearly from 52.4% in 2016 to 70.1% in 2020.^9^ Substantial exposure to antibiotics and unnecessary antibiotic prescribing among persons on maintenance hemodialysis has contributed to high rates of antibiotic-associated adverse events, including infections caused by antimicrobial-resistant bacteria and *C. difficile*.^10–13^ The need for antimicrobial stewardship programs in in-center dialysis facilities is therefore critical.

The development and implementation of effective antimicrobial stewardship programs, which target the unique characteristics of persons on maintenance hemodialysis and in-center dialysis settings, require detailed data on antibiotic prescribing patterns across facilities and an understanding of the variability in prescribing practices in order to identify targets for stewardship interventions; these data are limited.

To inform future stewardship initiatives and optimize antibiotic use in the dialysis population, a retrospective study among 16 in-center dialysis units in the United States was performed over a 3-year period, to quantify antibiotic prescribing trends and variability between facilities and, specific facility- and patient-level factors associated with antibiotic prescribing.

## Methods

### Study design and setting

A retrospective observational cohort study was performed examining antibiotic prescribing patterns across 16 in-center hemodialysis units over a 3-year period (April 1, 2021, to March 31, 2024). All participating dialysis units were operated by the same large dialysis organization and were located in urban settings in the New England area.

### Participants and variables

Adult patients (≥21 years old) receiving maintenance hemodialysis at participating facilities were included in the analysis. Patients were followed from their first dialysis session, within the study period, at any participating facility within the organization until the end of the study period, transfer to another facility outside of the 16 within the cohort, kidney transplantation, switch to peritoneal dialysis, or death. Individuals receiving peritoneal or home dialysis were excluded.

Demographic and clinical de-identified data were obtained from electronic health records of a large dialysis organization, including age, sex, race/ethnicity, primary insurance coverage, dialysis duration, etiology of end-stage renal disease (ESRD), type of vascular access, and comorbidities, using the Charlson Comorbidity Index (CCI).^14^ Data were aggregated at the facility level. Facilities were then categorized by aggregate antibiotic prescribing trends over the 3-year follow-up period, stratified by six-month increments. To examine patient- and facility-level characteristics associated with decreasing trends in antibiotic prescribing, facilities were grouped into two categories: Group 1 included those with a statistically significant decline in prescribing over the three-year period, and Group 2 included those without a significant decline.

Facility-level characteristics were obtained from the CMS Dialysis Facility Compare database as well as direct contact, including the number of dialysis stations, offering nocturnal hemodialysis, and academic affiliations.

The primary outcome variable was the rate of intravenous antibiotic doses prescribed per 100 patient-months, stratified by facility group. Secondary outcomes included rates of specific antibiotic prescribing, categorized by antibiotic type (vancomycin, cefazolin, cefepime, ceftazidime, daptomycin, ceftriaxone, aminoglycosides [gentamicin, tobramycin, amikacin], carbapenems [meropenem, ertapenem], and ceftazidime/avibactam) and hospitalization rates.

A new antibiotic course was defined as re-initiation of antibiotic therapy after at least 7 consecutive days without antibiotics. Course duration was defined as the number of days from the first to the last antibiotic administration within a course

### Statistical analysis

We analyzed antibiotic prescribing trends across the 16 dialysis units over the 3-year study period. The primary metric was antibiotic doses per 100 patient-months, calculated for each 6-month interval (six intervals total) for the overall cohort, for each individual dialysis unit, and for specific antibiotic types. Tests for trend were performed to assess changes in antibiotic prescribing over time using general linear models (GLM) with time as a continuous variable.

The 16 dialysis units were classified into two groups based on whether they demonstrated a statistically significant decrease in antibiotic prescribing over the study period: Group 1 (units with significant decrease) and Group 2 (units without significant decrease). Baseline patient characteristics were compared between the two facility groups using χ^2^ tests for categorical variables and t-tests or Wilcoxon rank-sum tests for continuous variables, as appropriate.

We compared antibiotic prescribing and demographics variables between Groups 1 and 2, examining both overall prescribing rates and rates of specific antibiotic types, using generalized estimating equations (GEE) with an unstructured correlation matrix to estimate population-averaged effects while accounting for correlations between repeated measures.^15^ GEE models were adjusted for race, sex, age, primary insurance, ESRD type, dialysis duration, and the Charlson Comorbidity Index. To evaluate the changes in the total number of antibiotic doses or average rate of hospitalizations per 100 individual-months, GLM was employed over the 3-year study period.^16^

All analyses were conducted using SAS version 9.4 (SAS Institute, Cary, NC). All statistical tests were two-sided, and a *P* value < .05 was considered statistically significant.

### Ethics

This study was determined exempt by the BrownHealth Institutional Review Board.

## Results

### Characteristics of 16 in-center dialysis units

Among the 16 enrolled in-center dialysis units, all were operated by the same large dialysis organization and were located in urban settings in the New England area. Four (25%) were affiliated with an academic medical center and 4 (25%) provided nocturnal dialysis. The average number of dialysis station was 24, ranging from 16–51.

### Trends in antibiotic prescribing rates among 16 in-center dialysis units

During the 3-year study period, 3549 unique persons received in-center dialysis of whom 937 (26.4%) received at least one dose of an intravenous antibiotic. The average number of antibiotic doses prescribed was 15.36 per 100 patient months. The four most common antibiotics prescribed were vancomycin (7.33 doses/100 patient months [48% of all antibiotic doses]), followed by cefazolin (3.9 doses/100 patient months [25% of all antibiotic doses]), cefepime (1.6 doses/100 patient months [10% of all antibiotic doses]) and ceftazidime (1.45 doses/100 patient months [9% of all doses]) (Table 1). Among antibiotic-exposed patients, the mean number of antibiotic courses per patient during the 3-year study period was 1.38 (SD 0.87, IQR 0, range 3), and the mean course duration was 10.17 days (median 7 days, SD 10.80, IQR 11, range 40 days).

**Table 1.** Trends in antibiotic doses/100 patient months by type of antibiotic for 16 in-center hemodialysis units Table 1 long description.

	Overall average (3-year period)	Study period						*P* for trend
Antibiotics		4/1/21–9/30/21	10/1/21–3/31/22	4/1/22–9/30/22	10/1/22–3/31/23	4/1/23–9/30/23	10/1/23–3/31/24	
**Any antibiotics**	15.36	19.53	17.90	14.70	13.33	13.23	13.47	<.001
**Vancomycin**	7.33	8.84	8.07	7.72	6.7	6.43	6.24	<.001
**Cefazolin**	3.9	5.47	4.10	3.36	3.53	3.78	3.17	.01
**Cefepime**	1.6	1.15	2.19	1.55	1.74	1.22	1.77	.74
**Ceftazidime**	1.45	2.54	2.00	1.39	.71	.76	1.3	.01
**Daptomycin**	.4	.48	.44	.04	.26	.65	.54	.5
Aminoglycosides^*^	.29	.48	.68	.24	.18	.05	.09	.01
**Ceftriaxone**	.22	.2	.22	.38	.21	.24	.07	.74
Carbapenems^**^	.14	.31	.13	0	0	.1	.29	.3
**Ceftazidime/avibactam**	.03	.06	.08	.03	0	0	0	.21

* Aminoglycosides: gentamicin, tobramycin and amikacin.

** Carbapenems: meropenem and ertapenem.

Rates of antibiotic doses prescribed decreased significantly over the study period when assessed across all six 6-month intervals (*P* < .001). Although some interval-to-interval variability was observed, the overall temporal trend was downward. The prescribing rate was 19.53 doses per 100 patient-months in the first 6-month interval and 13.47 doses per 100 patient-months in the final 6-month interval. Overall, there was a 34.66% decrease in antibiotic doses prescribed between these two intervals (377.0 doses/1930.36 patient months vs 246.33 doses/1828.76 patient months).

Among specific types of antibiotics, significant decreases were observed in the prescribing rates of several antibiotics. Vancomycin prescribing decreased from 8.84 to 6.24 doses/100 patient-months (170.67 doses/1930.62 patient months vs 114.33 doses/1832.26 patient months, 33.0% reduction, *P* < .001). Cefazolin prescribing decreased from 5.47 to 3.17 doses/100 patient-months (105.5 doses/1928.70 patient months vs 58.0 doses/1832.27 patient months, 45.0% reduction, *P* = .01), ceftazidime prescribing decreased from 2.54 to 1.3 doses/100 patient months (49.0 doses/1929.13 patient months vs 23.83 doses/1833.33 patient months, 51.36% reduction, *P* = .01) and aminoglycosides decreased from 0.48 to 0.09 doses/100 patient months (9.17 doses/1909.72 patient months vs 1.5 doses/1666.67 patient months, 83.64% reduction, *P* = .01). Other antibiotics showed nonsignificant trends toward decreased use (Table 1).

### Trends in rates and patterns of antibiotic prescribing among individual facilities

The average rates of antibiotic prescribing during the study period varied substantially among different units ranging from 4.82 doses/100 patient months to 30.87 doses/100 patient months. Three units had overall average antibiotic use exceeding 20 doses/100 patient-months, while six units had averages below 11 doses/100 patient-months. Among the 16 units, six (37.5%) demonstrated a statistically significant decrease in antibiotic prescribing over the study period (Table 2).

**Table 2.** Trends in all antimicrobial doses/100 patient months by individual units

		Study period						
Units	Overall average (3-year period)	4/1/21–9/30/21	10/1/21–3/31/22	4/1/22–9/30/22	10/1/22–3/31/23	4/1/23–9/30/23	10/1/23–3/31/24	*P* for trend
**A**	30.87	39.83	36.04	25.79	25.99	33.17	24.42	.04
**B**	24.07	32.59	26.21	19.56	20.2	22.1	23.75	.03
**C**	20.16	42.2	22.25	7.17	17.52	12.76	19.06	.03
**D**	17.34	11.15	12.8	21.36	27.19	10.86	20.7	.09
**E**	15.15	19.68	15.49	25.1	11.12	7.59	11.94	.18
**F**	14.47	12.07	14.36	13.41	17.28	15.97	13.73	.53
**G**	14.11	16.14	24.8	14.87	9.53	9.84	9.49	.01
**H**	13.09	24.55	14.95	13.58	7.82	11.71	5.94	.01
**I**	12.5	10.74	15.74	8.11	7.37	12.24	20.8	.18
**J**	11.37	15.46	16.85	9.0	12.83	5.59	8.48	.1
**K**	10.99	14.34	10.43	16.27	6.2	10.78	7.93	.14
**L**	10.84	14.65	10.37	7.8	10.3	11.5	10.44	.22
**M**	10.7	12.5	21.24	8.03	4.69	8.37	9.36	.1
**N**	10.7	9.96	12.93	3.6	12.46	13.97	11.25	.45
**O**	8.45	10.72	8.77	15.49	5.0	6.52	4.18	.03
**P**	4.82	2.69	4.18	6.54	5.08	4.67	5.76	.57

**Figure 1.**
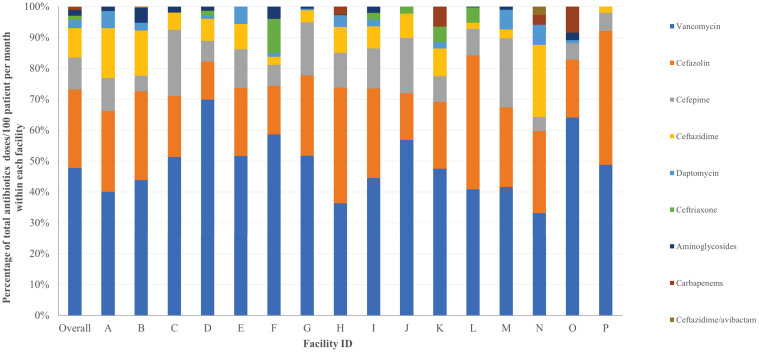
Percentages of antibiotic doses/100 patient months by antibiotic type for individual units.

There was a substantial range in the frequency of types of antibiotics prescribed among all doses between units; among the four most commonly prescribed antibiotics, vancomycin prescribing ranged from 33 to 70%, cefazolin from 12–43%, cefepime from 5–22% and ceftazidime from 0–23% (Figure 1). Although all units prescribed at least one dose of vancomycin, cefazolin and cefepime during the study period, several units did not prescribe any doses of the following antibiotics (number of units): carbapenems (12), ceftriaxone (10), aminoglycosides (8), and daptomycin (5).

### Comparisons between dialysis units with and without a decrease in antibiotic prescribing

#### Trends in antibiotic prescribing and hospitalizations

Table 3 compares trends over the 3-year study period between the 6 dialysis units showing a statistically significant decrease in antibiotic prescribing (Group 1) and the 10 dialysis units that did not show a decrease in prescribing (Group 2). Among Group 1, there were significant decreases in vancomycin, cefazolin, ceftazidime and aminoglycosides (*P* ≤.05). In Group 2, no individual antibiotic levels decreased.

**Table 3.** Trends in antimicrobial doses/100 patient months by type comparing six in-center dialysis units with a decrease in antibiotic prescribing (Group 1) and 12 in-center dialysis units without a decrease in antibiotic prescribing (Group 2) Table 3 long description.

Antibiotics	Overall average (3-year period)	Study period						*P* for trend
		4/1/21–9/30/21	10/1/21–3/31/22	4/1/22–9/30/22	10/1/22–3/31/23	4/1/23–9/30/23	10/1/23–3/31/24	
**All antibiotics**								
Group 1	19.69	29.36	24.25	17.49	15.18	16.82	15.07	<.001
Group 2	12.49	12.81	14.06	12.93	12.07	10.64	12.42	.18
**Vancomycin**								
Group 1	8.56	12.26	10.62	7.7	6.81	7.4	6.54	<.001
Group 2	6.4	6.2	6.14	7.73	6.6	5.73	6.03	.55
**Cefazolin**								
Group 1	5.31	8.51	5.9	4.57	4.89	5.88	2.1	.01
Group 2	2.82	3.14	2.72	2.4	2.49	2.22	3.94	.62
**Cefepime**								
Group 1	2.03	1.21	2.24	2.41	2.28	1.62	2.42	.9
Group 2	1.28	1.12	2.14	.88	1.34	.92	1.29	.24
**Ceftazidime**								
Group 1	2.14	4.58	2.4	1.64	.61	1.24	2.39	.04
Group 2	.92	.97	1.68	1.19	.78	.41	.51	.07
**Daptomycin**								
Group 1	.52	.52	.87	.08	.29	.59	.78	.68
Group 2	.31	.45	.11	0	.24	.69	.36	.31
**Ceftriaxone**								
Group 1	0	0	0	0	0	.02	0	–
Group 2	.38	.35	.38	.67	.37	.41	.11	.69
**Aminoglycosides**								
Group 1	.48	.95	1.05	.51	.15	.04	.17	.01
Group 2	.14	.11	.40	.03	.21	.05	.02	.47
**Carbapenems**								
Group 1	.15	.37	.00	0	0	0	.5	.23
Group 2	.13	.26	.23	0	0	.16	.14	.9
**Ceftazidime/avibactam**								
Group 1	.03	0	.12	.06	0	0	0	–
Group 2	.03	.11	.05	0	0	0	0	–

During the study period, there was no difference in the average rate of hospitalizations per 100 patient months between Groups 1 and 2 (15.79 versus 17.09, *P* = .73). There was also no difference in the trend of rates of hospitalizations (Group 1 *P* = .79, Group 2 *P* = .13).

#### Unit-level factors

There were no significant differences in unit-level factors between Group 1 and 2. All units were affiliated with a large dialysis organization and in an urban settings. There were no differences between Groups 1 and 2 for the following factors: affiliation with an academic center (2 [33%] and 2 [20%], *P* = .6), average number of stations (28.8 [range 16–51] and 20.4 [range 17–27], *P* = .18), or nocturnal dialysis shift (2 [33%] and 2 [20%], *P* = .6).

In 2018, an Antibiotic Stewardship protocol was implemented by the large dialysis organization among all in-center dialysis units. This protocol assisted prescribers in identifying and initiating empiric therapy in patients with suspected bloodstream infections (BSI), in guiding antibiotic management of patients with confirmed BSI, and in discontinuing antibiotics for those patients in whom there was no evidence of a BSI. Protocol utilization was not captured as part of the data collection process. In addition, a separate Antimicrobial Stewardship educational intervention was implemented in May, 2022, which provided all prescribers in 8 of the 16 study dialysis units with algorithms focusing on de-escalation of vancomycin to cefazolin for methicillin-susceptible *Staphylococcus aureus* infections and de-escalation of broad-spectrum cephalosporins to cefazolin for cefazolin-susceptible infections caused by gram-negative bacteria. Compliance with this educational intervention was not available. Among these 8 dialysis units, one was in Group 1 (16.7%) and 7 (70%) were in group 2 (*P* = .12).

#### Patient-level factors

Among the 3549 unique persons, 106 received in-center hemodialysis at both the Group 1 and 2 units (total 3,655). Table 4 presents the results of the GEE model comparing patient-level factors between dialysis units in Groups 1 and 2. Several independent patient-level factors were more frequent among individuals in Group 1 compared to Group 2, including Black race, Hispanic ethnicity, age group 56–65 years old, Medicare insurance status and diabetes mellitus as the cause of their ESRD. Access type differed between groups. The descriptive proportion of central venous catheter use was lower in Group 1 than Group 2 (28.9% versus 33.4%), while arteriovenous fistula use was similar between groups (46.2% versus 46.0%). In the univariate GEE model, central venous catheter use was not significantly associated with Group 1 compared with arteriovenous fistula use (OR 0.87, 95% CI 0.36–2.13, *P* = .77). In the multivariable GEE model, central venous catheter use was modestly associated with Group 1 (adjusted OR 1.11, 95% CI 1.01–1.22, *P* = .03).

**Table 4.** Comparison of patient-level factors between six in-center dialysis units with a decrease in antibiotic prescribing (Group 1) and ten in-center dialysis units without a decrease in antibiotic prescribing (Group 2)

	Study groups					
	Group 1	Group 2	Univariate		Multivariable^*^	
Patient-level factors	N = 1,496	N = 2,159	OR [95% CI]	*P* value	OR [95%CI]	*P* value
**Age groups**						
16–30	23 (1.5)	36 (1.7)	.94 [.55–1.61]	.81	–	–
31–45	131 (8.8)	150 (6.9)	1.29 [.98–1.68]	.06	1.06 [.63–1.79]	.84
46–55	217 (14.5)	250 (11.6)	1.27 [1.02–1.59]	.03	1.20 [.81–1.78]	.37
56–65	368 (24.6)	461 (21.4)	1.16 [.96–1.4]	.12	1.34 [1.03–1.73]	.03
66–75	419 (28)	606 (28.1)	Reference			
76–85	259 (17.3)	498 (23.1)	.77 [.63–.94]	.01	.94 [.72–1.21]	.61
>85	79 (5.3)	158 (7.3)	.75 [.55–1.01]	.06	–	–
*Missing*	0 (.0)	0 (.0)	–	–	–	–
**Gender**						
Male	876 (58.6%)	1,287 (59.6%)	Reference			
Female	620 (41.4%)	872 (40.4%)	1.04 [.91–1.19]	.6	1.01 [.86–1.2]	.89
*Missing*	0 (.0)	0 (.0)	–	–	–	–
**Race/ethnicity**						
White	502 (35.4)	1,176 (60.2)	Reference			
Black	554 (39.1)	302 (15.5)	4.31 [3.61–5.14]	<.001	5.02 [4.07–6.21]	<.001
Asian	42 (3.)	93 (4.8)	1.09 [.75–1.6]	.65	–	–
Other	36 (2.5)	83 (4.3)	.99 [.66–1.49]	.9	1.07 [.67–1.71]	.78
Hispanic	284 (20)	301 (15.4)	2.25 [1.85–2.73]	<.001	2.55 [1.99–3.26]	<.001
*Missing*	78 (5.2)	204 (9.4)	–	–	–	–
**Primary insurance**						
Commercial	100 (6.8)	227 (10.6)	Reference			
Medicare	1,112 (75.1)	1632 (76.1)	1.59 [1.24–2.04]	<.001	1.46 [1.04–2.04]	.03
Medicaid	268 (18.1)	285 (13.3)	2.16 [1.61–2.9]	<.001	1.29 [.87–1.91]	.2
*Missing*	16 (1.1)	15 (.7)	–	–	–	–
**ESRD type**						
Hypertension	248 (20.7)	407 (25.1)	Reference			
DM	617 (51.4)	740 (45.6)	1.33 [1.1–1.61]	.01	1.36 [1.07–1.74]	.01
Cystic	37 (3.1)	46 (2.8)	1.38 [.86–2.21]	.18	–	–
Glomerular	118 (9.8)	179 (11.0)	1.05 [.79–1.4]	.73	1.21 [.87–1.66]	.25
Urologic	31 (2.6)	32 (2.0)	1.58 [.94–2.65]	.08	–	–
Other	150 (12.5)	220 (13.6)	.99 [.7–1.38]	.9	1.35 [1.00–1.83]	.05
*Missing*	295 (19.7)	535 (24.8)	–	–	–	–
**Access type**						
AVF (%)	11,293 (46.2)	15,100 (46.0)	Reference			
AVG (%)	6,070 (24.9)	6,756 (20.6)	.99 [.55–1.81]	.9	.95 [.77–1.18]	.67
CVC (%)	7,051 (28.9)	10,974 (33.4)	.87 [.36–2.13]	.77	1.11 [1.01–1.22]	.03
*Missing*	6 (.02)	17 (.05)	–	–	–	–
**Mean duration on dialysis > 4.1 years**						
	489 (34.6)	584 (28.5)	1.32 [1.14–1.53]	<.001	1 [.84–1.2]	.9
*Missing*	83 (5.5)	110 (5.1)	–	–	–	–
**Mean Charlson comorbidity (CCI) Index > 5.4**						
	733 (49.0)	1,136 (52.6)	.87 [.76–.99]	.02	.9 [.74–1.09]	.28
*Missing*	0 (.0)	0 (.0)	–	–	–	–

– indicates data not available or not determined.

* GEE model adjusted by race, gender, age, primary insurance, ESRD type, duration on dialysis, and CCI when applicable.

## Discussion

In this multicenter retrospective cohort study, trends in intravenous antibiotic prescribing patterns were quantified and characterized in 16 in-center hemodialysis units over a 3-year period. As opposed to the majority of previous studies in the population of dialysis, which focused only on intravenous antibiotic starts, all doses of prescribed antibiotics were analyzed.^9^ The overall rate of any antibiotic prescribed was 15.36/100 patient months. The most common antibiotics prescribed were vancomycin, cefazolin, ceftazidime and cefepime. Substantial heterogeneity in prescribing patterns between the 16 units were identified. Rates of antibiotic prescribing ranged from 4.82 to 30.87 doses/100 patient months. The overall percentage of types of antibiotics prescribed also varied considerably. Among the 16 units, vancomycin prescribing ranged from 33 to 70% of all antibiotic doses, cefazolin from 12–43%, cefepime from 5–22% and ceftazidime from 0–23%. Although all units prescribed at least one dose of vancomycin, cefazolin and cefepime during the study period, only a subset prescribed carbapenems, ceftriaxone or aminoglycosides.

Despite numerous educational interventions from both nephrology and infectious disease organizations, aimed at improving and thereby decreasing antibiotic exposure, antibiotic prescribing did not decrease in 10 (62.5%) of the 16 in-center hemodialysis units.^5,17^ These data suggest that further efforts in antimicrobial stewardship are needed in the hemodialysis population.

Among the 6 in-center hemodialysis units with a decrease in antibiotic prescribing, the antibiotics with a statistically significant decrease over the 3-year study period were vancomycin, cefazolin, ceftazidime and aminoglycosides. To identify factors potentially associated with reduced prescribing, the six units that showed a decrease in prescribing were compared with units that did not show a decrease. Several patient-level characteristics were identified, including differences in race and ethnicity, age, diabetes as the cause of ESRD, and Medicare insurance status. These observed associations are complex and warrant careful interpretation. Although CVC use was more common in Group 2 in unadjusted comparisons, consistent with the expectation that catheter use would be associated with an increase in antibiotic prescribing, the adjusted association reversed. This reversal likely reflects confounding by demographic and clinical characteristics or model instability from correlated covariates, rather than a clinically plausible association between CVC use and improved prescribing trends. Differences in unit-level factors including size and affiliation with an academic center were not identified. This study was retrospective and therefore only those factors that could be accurately obtained were included in the analyses. Other important factors that could have contributed to differences in prescribing rates need further study and include compliance with infection prevention and control policies as well as antimicrobial stewardship programs implemented during the study period. In addition, data on the clinical indications for antibiotic prescribing, types of infections, rates of antibiotic-resistant bacteria and number of prescribers were not evaluated.

The substantial heterogeneity in antibiotic prescribing, including trends, rates and types of antibiotics between these 16 units, emphasize the need for developing and implementing standardized antimicrobial stewardship programs which target the unique characteristics of in-center hemodialysis units. These effective programs, which are mandated in the hospital setting, provide guidance toward optimal antibiotic prescribing for empiric and directed infection treatment, duration and de-escalation once the pathogen is identified.^3,17^ In this study, facility-level characteristics that explained declining trends were not identified, including the distribution of educational material. This suggests that passive educational initiatives alone may be insufficient to change entrenched prescribing behaviors. Although it is well-established that educational interventions are necessary and effective, they are insufficient on their own to lead to practice change. Additional interventions to reduce antibiotic prescribing and influence clinician behavior include more active, data-driven feedback and prospective audits.^18^ Barriers and facilitators related to de-implementation science for reducing ineffective antibiotic use, a central element of stewardship programs, are outlined in two recent Society for Healthcare Epidemiology Research Committee White Papers.^19,20^

Antimicrobial stewardship programs have also been shown to significantly decrease inappropriate antibiotic prescribing in in-center dialysis settings and decrease the negative consequences of overprescribing.^5,21–23^ A decision analytic model on the clinical and economic consequences of implementing nationwide antimicrobial stewardship programs in in-center dialysis facilities predicted a 4.8% reduction in infections caused by multidrug-resistant bacteria and *Clostridioides difficile*; a 4.6% reduction in infection-related deaths and a cost savings of $106,893,517 (5.0% reduction) per year.^21^ These data emphasize the need to develop effective antimicrobial stewardship programs in out patient dialysis facilities. In this study, several factors were identified among units with a significant decrease in antibiotic prescribing and provides initial guidance on developing programs targeting the in-center dialysis settings. Importantly, among the 6 units that demonstrated a decreasing trend in prescribing, an increase in hospitalizations was not detected, suggesting an absence of negative outcomes.

This analysis has several limitations. First, the available database did not include oral antibiotics. As a result, it was not possible to assess whether switching from intravenous to oral antibiotics contributed to the observed reduction in antibiotic use across the six dialysis units. Second, the numerous educational initiatives toward improving antibiotic prescribing in out patient dialysis units, provided by national organizations, were not captured in this study and likely contributed to the decreasing trend. Third, because prescribing rates were summarized in 6-month intervals, seasonal effects may have contributed to interval-to-interval variation. However, the primary inference was based on the overall trend across all study intervals. Lastly, the study included units from the New England area and results may not be applicable to other regions.

This study provides comprehensive data to support the need for antimicrobial stewardship programs in the out patient dialysis setting. These results may inform the development and implementation of future effective programs in this setting that will ultimately improve the outcomes of persons requiring maintenance hemodialysis.

## Table 1. Long description

The table presents data on antibiotic doses per 100 patient months by type of antibiotic for 16 in-center hemodialysis units over a 3-year study period. It includes an overall average for the 3-year period and specific study periods. The table has 13 rows and 9 columns. The columns are labeled as follows: Overall average (3-year period), 4/1/21-9/30/21, 10/1/21-3/31/22, 4/1/22-9/30/22, 10/1/22-3/31/23, 4/1/23-9/30/23, 10/1/23-3/31/24, and P for trend. The rows are labeled with different types of antibiotics: Any antibiotics, Vancomycin, Cefazolin, Cefepime, Ceftazidime, Daptomycin, Aminoglycosides, Ceftriaxone, Carbapenems, and Ceftazidime/avibactam. Each cell contains the number of doses per 100 patient months for the corresponding antibiotic and study period. Notable trends include a general decrease in the use of most antibiotics over the study period, with significant P values for trend indicating statistical significance in the changes observed.

Navigate back to Table 1..

## Table 3. Long description

The table compares antibiotic prescribing trends over a 3-year study period between two groups of dialysis units. Group 1 consists of six units showing a decrease in antibiotic prescribing, and Group 2 consists of ten units without a decrease. The table has 12 rows and 8 columns. The columns are labeled Overall average (3-year period), 4/1/21-9/30/21, 10/1/21-3/31/22, 4/1/22-9/30/22, 10/1/22-3/31/23, 4/1/23-9/30/23, 10/1/23-3/31/24, and P for trend. The rows are labeled with different antibiotics: All antibiotics, Vancomycin, Cefazolin, Cefepime, Ceftazidime, Daptomycin, Ceftriaxone, Aminoglycosides, Carbapenems, and Ceftazidime/avibactam. Each antibiotic is further divided into Group 1 and Group 2. The values in the table represent the doses per 100 patient months for each antibiotic in the respective groups and time periods. Notable trends include significant decreases in vancomycin, cefazolin, ceftazidime, and aminoglycosides in Group 1, while no individual antibiotic levels decreased in Group 2.

Navigate back to Table 3..
